# Association between Menopausal Symptoms and Overactive Bladder: A Cross-Sectional Questionnaire Survey in China

**DOI:** 10.1371/journal.pone.0139599

**Published:** 2015-10-08

**Authors:** Lingping Zhu, Xiaoxia Cheng, Jiaxin Sun, Shiyi Lv, Suzhen Mei, Xing Chen, Sisi Xi, Jin Zhang, Mukun Yang, Wenpei Bai, Xiaoyan Yan

**Affiliations:** 1 Department of Obstetrics and Gynecology, Peking University First Hospital, Beijing, China; 2 Department of Gynecology and Obstetrics, The Ninth People's Hospital, Shenzhen, Guangdong Province, China; 3 Department of Gynecology and Obstetrics, Second Hospital of Sanming City, Fujian Province, China; 4 Peking University Clinical Research Institute, Beijing, China; BRAC, BANGLADESH

## Abstract

**Purpose:**

The association between menopause and overactive bladder is controversial. The purpose of this study was to determine the association between menopausal symptoms and overactive bladder, and identify the risk factors for overactive bladder.

**Methods:**

A cross-sectional study was performed. The study included 403 women aged 36–76 years who visited the menopause clinic at Peking University First Hospital between September 2012 and December 2013. The overactive bladder symptom score and modified Kupperman index questionnaires were used. Differences were assessed using descriptive statistics to determine any association between the overactive bladder symptom score and modified Kupperman index score, and to evaluate the risk factors for overactive bladder.

**Results:**

A total of 304 women were finally enrolled. The prevalence of overactive bladder was 9.43%, and the modified Kupperman index score; number of sexual problems; and frequency of urinary tract infections, vertigo, melancholia, and mood swings were significantly higher in patients with overactive bladder than in the patients without overactive bladder (p < 0.05). Menopausal symptoms (modified Kupperman index score ≥ 15) (odds ratio: 1.049, 95% confidence interval: 1.006–1.095, p = 0.025) and a low frequency of sexual intercourse in the last 6 months (odds ratio: 2.580, 95% confidence interval: 1.228–5.422, p = 0.012) were identified as independent risk factors for overactive bladder. The frequency of sexual intercourse was found to decrease with an increase in the severity of overactive bladder (p = 0.004, linear-by-linear association = 0.001).

**Conclusion:**

Menopausal symptoms may be closely associated with overactive bladder, and sexual activity may be associated with the severity of overactive bladder. Moreover, sexual problems, urinary tract infections, vertigo, melancholia, and mood swings may be associated with overactive bladder.

## Introduction

With the gradual aging of China’s population, health problems specific to the elderly, especially chronic conditions such as overactive bladder (OAB), are presenting new challenges to the Chinese healthcare system. The prevalence of OAB has been reported to be 10–15% [1]. Urological symptoms and their emotional burden have a significant negative impact on patients with OAB, especially those with OAB wet type involving urge incontinence [2]. OAB has also been reported to adversely affect the social life of a patient [3].

OAB is a clinical condition and has been defined by the International Continence Society as the presence of “urinary urgency, usually accompanied by frequency and nocturia, with or without urge urinary incontinence (UI), in the absence of a urinary tract infection (UTI) or other obvious pathology” [4]. A recent survey showed an increase in the prevalence of OAB in individuals aged 40–50 years, and the association between age and OAB has been confirmed [5]. Menopausal syndrome refers to a series of physical and psychological symptoms in perimenopause and post-menopause because of the fluctuation of hormone levels [6]. Additionally, menopausal symptoms are not universal and can be affected by culture, race, and ethnicity [7]. Natural female menopause usually occurs between 45–55 years of age worldwide according to the World Health Organization report in1996. This report also points out that urinary problems are common in perimenopause, and estrogen therapy may improve some symptoms. This led us to hypothesize that there may be an association between menopausal syndrome and OAB. However, this association is controversial. In daily clinical practice, we found that OAB was more likely to develop in women with severe menopausal symptoms than in those without menopausal symptoms; therefore, we conducted a cross-sectional study to determine the association between menopausal symptoms and OAB, and identify the risk factors for OAB. To our knowledge, this is the first study of the world to use the overactive bladder symptom score (OABSS) and the modified Kupperman index (mKMI) to evaluate the association between menopausal symptoms and OAB in women with menopausal symptoms.

## Materials and Methods

### Participants

The study included 403 women aged 36–76 years visiting the menopause clinic of Peking University First Hospital between September 2012 and December 2013. The Peking University First Hospital is a famous Third Class A hospital in China, with an average of 7000 consultations per day in all its outpatient clinics. About half of its patients are from other provinces in China. The menopause clinic is an outpatient clinic of this hospital. All of the women provided consent to participate in the study, and the study was approved by the Clinical Ethics Committee of Peking University First Hospital (Grant No. 2013 [681]).

### Criteria

Patients who visited the menopause clinic with menopausal symptoms were included. Patients with urological abnormalities, UTI (including urocystitis), neurological or severe psychiatric diseases, diabetic bladder, or pelvic organ prolapse, and those who underwent urogenital procedures or were treated for both menopausal symptoms and OAB or other related diseases (using diuretics) were excluded. A total of 304 women were finally enrolled in the study. Participants were classified as having either natural menopause or menopausal symptoms. WHO defines natural menopause as the permanent cessation of menstruation resulting from the loss of ovarian follicular activity. Natural menopause is recognized to have occurred after 12 consecutive months of amenorrhea for which there is no other obvious pathological or physiological cause [8]. Menopausal symptoms refer to a wide variety of physical and emotional experiences that may or may not be related to hormone or menstrual changes, including hot flashes, vaginal dryness, loss of libido, depression, anxiety, irritability, poor memory, loss of concentration, mood swings, insomnia, tiredness, aching limbs, loss of energy, and dry skin [9].

### Study design

**Fig 1:**
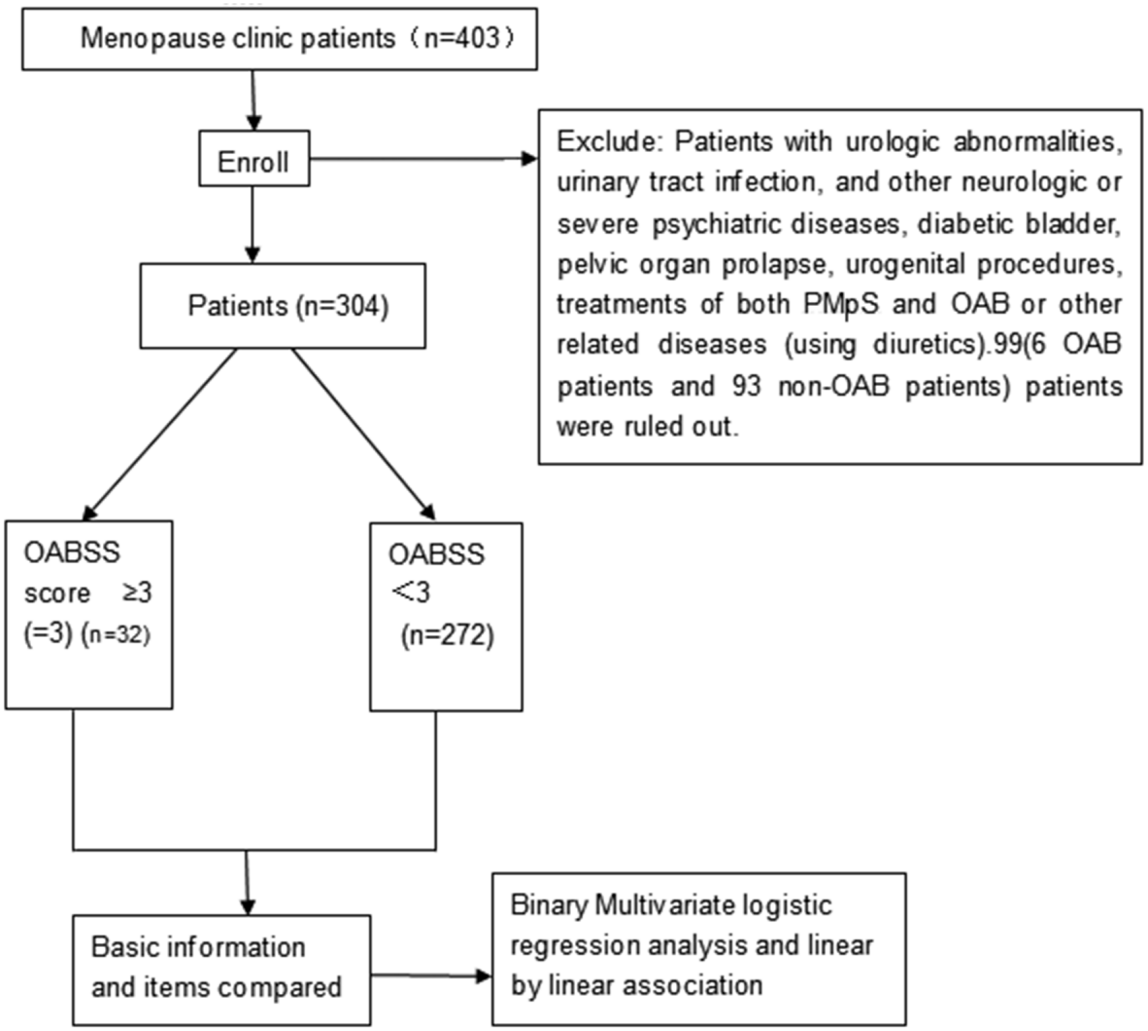
The study flow diagram of this survey, 99 patients were excluded according to the exclusion criteria, 92 patients were Non-OAB patients, 6 patients were OAB patients.

A cross-sectional questionnaire survey was administered to the women enrolled in the study. Fig 1 presents the study flow diagram.

### Questionnaire

A self-administered questionnaire comprising 260 items over five pages was designed, and the women completed it in approximately 20 minutes. The questionnaire recorded general information (age, profession, body weight, body mass index [BMI], marital status, educational level, menstrual history, childbearing history, and previous or current diseases based on diagnoses from the patient’s doctor or during annual physical examinations). The mKMI, which is widely used to evaluate menopausal symptoms, was also included in the questionnaire. The OABSS is widely used to diagnose OAB, drug use, and lifestyle. The patient’s attitude towards the occurrence of OAB was also assessed through the questionnaire. Most of the independent variables, such as menopausal status, marital status, profession, educational background, exercise times, monthly income, complications (according to medical records), and the mKMI and OABSS results were investigated using closed-ended questions, while other variables, including age at menarche, last menstrual period, duration of menopausal symptoms, height, and weight, were assessed using open-ended questions. All women completed the questionnaire under the guidance of doctors who were trained and certified in the use of the questionnaire. Trained doctors filled in the items in the questionnaire that needed be calculated, such as BMI, mKMI, and OABSS, and the other items were filled in by the patients. Trained doctors interpreted medical terms in the questionnaire. All doctors were from the Department of Obstetrics and Gynecology. The time to fill out the questionnaire was restricted to 20 minutes, after which trained doctors collected and sorted the information from the collected questionnaires.

### OAB and menopause evolution

OAB was diagnosed using the OABSS, which is a symptom assessment tool designed to quantify OAB symptoms into a single score. It includes questions about the following four symptoms of OAB: daytime frequency, nighttime frequency, urgency, and urge incontinence. The Chinese version of the OABSS scale has been validated for the Chinese population [10].

The subjects were asked to rate their symptom severity using a Likert scale, with maximum (most severe) scores of 2, 3, 5, and 5 for daytime frequency, nighttime frequency, urgency, and urge incontinence, respectively. The total score ranged from 0 to 15, with a higher score indicating more severe OAB. According to clinical guidelines, a minimum OABSS of 3 and a minimum urgency score of 2 are required to diagnose OAB, and the severity of OAB is classified as mild (OABSS, 3–5), moderate (OABSS, 6–11), or severe (OABSS, 12–15) [11].

The mKMI is more sensitive than the menopause rating scale for the evaluation of menopausal symptoms in Chinese populations [12], and it considers 13 symptoms divided into the following four grades (0–3 points) according to severity: 0, no symptoms; 1, mild symptoms; 2, moderate symptoms; and 3, severe symptoms. The weighted score for hot flashes or sweating is 4 points; paresthesia, insomnia, mood swings, sexual problems, and urinary infection are scored 2 points each; and other symptoms are scored 1 point each. We calculated the morbidity of menopausal symptoms using an mKMI cutoff score of ≥ 15, which was the diagnostic criterion. Menopausal symptoms were classified according to mKMI scores as mild (mKMI scores of 15–24 points), moderate (mKMI scores of 25–34), or severe (mKMI scores of ≥ 35). All of the above criteria were based on definitions cited in the *Journal of Chinese Gynecology and Obstetrics*, 2007 [6].

### Study size

This was a cross-sectional study. PASS 11 software (NCSS Inc., Kaysville, UT) was used to determine the study size. The sample of this study was 403 patients. To determine the study size, we used the prevalence of OAB, where p = 0.088 [13], and at a 0.05 significance level. According to the exclusion criteria, 304 patients were included, and the power of the test was 78.3%.

### Statistical analysis

Statistical analyses were performed using a Statistical Analysis System (v.9.4; SAS Institute Inc., Cary, NC). Data are presented as means ± standard deviations (SDs) for continuous variables or as numbers for age, menarche, final menstrual period, and weight. The median, mean, maximum, and minimum, Q1, and Q3 values were calculated for final menstrual period and duration of menopausal symptoms. Categorical variables were expressed as frequencies and percentages. The chi-square test and Fisher’s exact test were used to evaluate differences for categorical variables. The differences between OAB and non-OAB patients were analyzed to explore any association between OABSS and KMI scores and potential risk for OAB. Spearman correlation was performed before we set the binary logistic regression model to eliminate the possibility of collinearity among variables. Binary multivariate logistic regression analysis (method = ENTER) was used to identify the factors associated with OAB, after adjusting for age, coronary artery disease (CAD), diabetes mellitus (DM), and BMI. A linear-by-linear association test was used to evaluate the association between OAB and potential risk factors. Odds ratios (ORs) and 95% confidence intervals (CIs) were used to evaluate the risk factors in both groups. A p-value of <0.05 was considered statistically significant.

## Results

### The general characteristics of the sample

Of the 403 questionnaires received, 99 were excluded according to the exclusion criteria. Therefore, 304 patients were included in the statistical analysis. Of these, 32 patients had OAB. The patients’ average age was 51.65±6.00 years. The educational level of the patients was as follows: undergraduate degree (103, 33.88%) junior college degree (77, 25.33%), and other degree (124, 40.79%). In terms of profession, they were state officers (94, 30.92%), professionals (75, 24.67%), or others (135, 44.41%). The patients’ average final menstrual period age was 48.81±5.07 years. The patients’ average menarche age was 13.69±1.63 years. The patients’ average BMI was 24.07±3.367. The patients’ average waistline measurement was 81.45±8.30 cm. The patients’ average weight was 61.60±9.06 kg. The patients’ average mKMI score was 19.42±9.66. The patient average OABSS score was 1.98±2.2.

### Separated participant characteristics

The number of patients without OAB was 272. The average age of the OAB patients was 52.06±5.725 years. The average age of the non-OAB patients was 51.60±6.034 years. No significant differences were noted in age, profession, weight, BMI, menarche, final menstrual period, duration of menopausal symptoms, or number of pregnancies, or in the presence of hypertension, DM, osteoporosis, hyperlipidemia, coronary heart disease, stroke, cervical spondylosis, uterine (myoma) fibroids, or benign ovarian tumor between the OAB and non-OAB patients (p > 0.05) (Table 1). There were no differences in basic information between the two groups.

**Table 1:** A comparison of patient characteristics of OAB and non-OAB patients.

Item	Index	OAB	Non-OAB	Total	Statistical magnitude	P
Age (years)	n (missing)	32 (0)	272 (0)	304 (0)	*t*-test	0.637
	Mean (SD)	52.06 (5.725)	51.60 (6.034)	51.648 (5.998)	0.472	
	Median	52.00	51.00	51.00		
Profession	State organizations, party and mass organizations, enterprises and institutions, n (%)	3 (9.68%)	91 (33.10%)	94 (31.54%)	Fisher’s exact test	0.071
	Professionals, n (%)	12 (38.7%)	63 (23.20%)	75 (25.17%)		
	Staff and related personnel, n (%)	5 (16.12%)	29 (10.70%)	34 (11.41%)		
	Commercial, service personnel, n (%)	3 (9.68%)	21 (7.70%)	24 (8.05%)		
	Agriculture, forestry, animal husbandry and fishery, water conservation, production personnel, n (%)	1 (3.23%)	4 (1.50%)	5 (1.68%)		
	Production, transportation equipment operators, and related personnel, n (%)	0 (0.00%)	1 (0.40%)	1 (0.34%)		
	Soldier, n (%)	0 (0.00%)	1 (0.40%)	1 (0.34%)		
	Other, n (%)	7 (22.58%)	57 (21.00%)	64 (21.48%)		
	Total	31	267	298		
Weight	n (missing)	32 (0)	272 (0)	94 (0)	*t*-test	0.161
	Mean (SD)	63.71 (10.01)	61.35 (8.83)	62.615 (9.76)	1.404	
	Median	63	60.85	62.00		
	Total	32	272	94		
BMI	< 19 (underweight)	0 (0.00%)	5 (1.84%)	5 (1.64%)	Fisher’s exact test	0.383
	19–24 (normal)	12 (37.50%)	123 (45.22%)	135 (44.41%)		
	24–29 (overweight)	16 (50%)	120 (44.12%)	136 (44.74%)		
	> 29 (obese)	4 (12.5%)	24 (8.82%)	28 (9.21%)		
	Total	32	272	304		
Menarche	n (missing)	32 (0)	272 (0)	94 (0)	*t*-test	0.968
	Mean (SD)	13.34 (1.73)	13.74 (1.62)	13.445 (1.65)		
	Median	13.00	14.00	13.00		
Final menstrual period	n (missing)	31 (1)	265 (7)	296 (8)	*t*-test	0.944
	Mean (SD)	48.84 (6.14)	48.00 (4.39)	48.82 (5.07)		
	Median	50.00	49	49.00		
	Q1, Q3	48.00, 52.00	46, 51	46.00, 51.00		
	Min, Max	25.00, 58.00	32.00, 71.00	25.00, 71.00		
Duration of menopausal symptoms	n (missing)	31 (1)	268 (4)	299 (5)	*F* test	0.248
	Mean (SD)	3.45 (4.78)	3.18 (3.328)	2.56 (3.851)		
	Median	1	2	1		
	Q1, Q3	0, 5	2, 4	0, 3		
	Min, Max	0, 16	0, 28	0, 28		
Number of pregnancies	0	3 (9.375%)	13 (4.80%)	16 (5.26%)	Fisher’s exact test	0.120
	1	4 (12.50%)	60 (22.10%)	64 (21.05%)		
	2	15 (46.875%)	100 (36.80%)	115 (37.83%)		
	≥ 3	10 (31.25%)	99 (36.30%)	109 (35.86%)		
	Total	32	272	304		
Hypertension	Yes	2 (6.25%)	42 (15.44%)	44 (14.47%)	Fisher’s exact test	0.062
	No	30 (93.75%)	230 (84.56%)	260 (85.53%)		
	Total	32	272	304		
Diabetes mellitus	Yes	2 (6.25%)	13 (3.33%)	15 (4.93%)	Fisher’s exact test	0.664
	No	30 (93.75%)	259 (96.67%)	289 (95.07%)		
	Total	32	272	304		
Osteoporosis	Yes	8 (25%)	41 (15.10%)	49 (16.12%)	χ² test	0.067
	No	24 (75%)	231 (84.90%)	255 (83.88%)		
	Total	32	272	304		
Hyperlipidemia	Yes	8 (25%)	59 (21.70%)	67 (22.04%)	χ² test	0.981
	No	24 (75%)	213 (78.30%)	237 (77.96%)		
	Total	32	272	304		
Coronary heart disease	Yes	1 (3.12%)	8 (2.94%)	9 (2.96%)	Fisher’s exact test	1.000
	No	31 (96.88%)	264 (97.06%)	295 (97.04%)		
	Total	32	272	304		
Stroke	Yes	0 (0%)	3 (1.10%)	3 (0.99%)	Fisher’s exact test	1.000
	No	32 (100%)	269 (98.90%)	301 (99.01%)		
	Total	32	272	304		
Cervical spondylosis	Yes	15 (46.88%)	118 (43.40%)	133 (43.75%)	χ² test	0.968
	No	17 (53.12%)	154 (56.60%)	171 (56.25%)		
	Total	32	272	304		
Uterine (myoma) Fibroid	Yes	11 (34.38%)	111 (40.80%)	122 (32.98%)	χ² test	0.718
	No	21 (65.62%)	161 (59.20%)	282 (67.02%)		
	Total	32	272	304		
Benign ovarian tumor	Yes	2 (6.25%)	24 (8.80%)	26 (8.55%)	Fisher’s exact test	0.493
	No	30 (93.75%)	248 (91.20%)	278 (91.45%)		
	Total	32	272	304		

OAB: overactive bladder; SD: standard deviation; Number of pregnancies: number of instances of a gravid uterus regardless of the outcome of pregnancy

We compared the following between groups:Hot flashes, Formication, Palpitations, Muscle/joint pain, Fatigue, Insomnia, Paresthesia, duration of menopausal symptoms, monthly income, exercise > 3 times weekly showed no significance in two groups. The mean mKMI score, frequency of sexual intercourse in the past 6 months, sexual problems, and frequency of UTI, vertigo, melancholia, and mood swings, these 7 items were significantly higher in the OAB patients than in the non-OAB patients (p < 0.05; Table 2). Additionally, the quality of life, measured according to the frequency of sexual intercourse over the past 6 months, was significantly lower in the OAB patients than in the non-OAB patients (p = 0.003; Table 2).

**Table 2:** A comparison of mKMI items and quality of life in OAB and non-OAB patients.

Item	Mean ± SD		P value (t-test)
	OAB	Non-OAB	
Hot flashes/sweating	1.06 ± 0.840	0.94 ± 0.897	0.466
Urinary tract infection	1.09 ± 1.174	0.60 ± 0.901	0.005
Sexual problems	1.41 ± 1.160	1.00 ± 1.007	0.035
Formication	0.50 ± 0.672	0.35 ± 0.637	0.213
Palpitations	1.13 ± 0.833	0.89 ± 0.864	0.138
Headaches	0.81 ± 0.738	0.75 ± 0.780	0.681
Muscle/joint pain	1.53 ± 0.879	1.26 ± 0.915	0.110
Fatigue	1.53 ± 0.718	1.35 ± 0.834	0.241
Vertigo	1.03 ± 0.861	0.69 ± 0.797	0.025
Melancholia	1.03 ± 0.740	0.73 ± 0.836	0.035
Mood swings	1.41 ± 0.837	1.04 ± 0.788	0.013
Insomnia	1.22 ± 0.792	1.15 ± 0.927	0.677
Paresthesia	1.03 ± 0.861	0.75 ± 0.814	0.066
mKMI score	24.13 ± 8.534	18.86 ± 9.648	0.003
Frequency of sexual intercourse in the past 6 months	3.66 ± 0.553	3.23 ± 0.745	0.003
Duration of menopausal symptoms	4.13 ± 4.390	3.18 ± 3.328	0.146
Monthly income	2.87 ± 0.990	2.66 ± 0.948	0.432
Exercise > 3 times weekly	1.38 ± 0.492	1.34 ± 0.474	0.690

KMI: Kupperman Index, mKMI: modified Kupperman index; OAB: overactive bladder

### Risk factors for OAB

Binary multivariate logistic regression analysis was performed to evaluate age, BMI, DM, CAD, frequency of sexual intercourse, and mKMI score. This identified menopausal symptoms (mKMI score ≥ 15) (OR: 1.049, 95% CI: 1.006–1.095, p = 0.025) and low frequency of sexual intercourse in the last 6 months (OR: 2.580, 95% CI: 1.228–5.422, p = 0.0012) as independent risk factors for OAB. However, age (p = 0.419), DM (p = 0.200), CAD (p = 0.862), and BMI (p = 0.528) were not identified as risk factors for OAB (Table 3).

**Table 3:** Multivariate analysis of parameters associated with OAB.

Variables	Adjusted OR (95% CI)	P
Age	0.970 (0.901–1.044)	0.419
Diabetes mellitus	0.331 (0.061–1.794)	0.200
CAD	1.224 (0.125–11.975)	0.862
Sexual intercourse*	2.580 (1.228–5.422)	0.012
mKMI*	1.049 (1.006–1.095)	0.025
BMI	1.038 (0.924–1.166)	0.528

Bivariate logistic regression analysis was used,

* mean p <0.05

BMI: body mass index; CAD: coronary artery disease; CI: confidence interval, mKMI: modified Kupperman index; OAB: overactive bladder; OR: odds ratio

### Association between sexual intercourse and OAB

Based on the findings presented in Table 3, the association between the frequency of sexual intercourse and OAB was evaluated. The results of a linear-by-linear association test showed that the frequency of sexual intercourse per month decreased as the severity of OAB increased (p = 0.004, linear-by-linear association = 0.001; Table 4).

**Table 4:** Association between sexual intercourse and OAB.

Sexual intercourse per month	None	≤ 3 times	4–7 times	≥ 8 times	P (Fisher’s exact test)	Linear-by-linear association
OAB (n = 32)	23	8	1	0		
Non-OAB (n = 272)	104	128	36	4	0.004	0.001

Fisher’s exact test and linear-by-linear association were used.

OAB: overactive bladder.

According to results shown above, menopausal symptoms and frequency of sexual intercourse were possibly independent risk factors of OAB, and statistically the frequency of sexual intercourse decreased as the severity of OAB increased.

## Discussion

In the present study, menopausal symptoms were identified as an independent risk factor for OAB. Additionally, the mKMI score, number of sexual problems, and frequencies of UTI, vertigo, melancholia, and mood swings were significantly higher in women with OAB than in patients without OAB. Moreover, the frequency of sexual intercourse over the past 6 months was found to be associated with OAB.

There is limited evidence of the association between menopausal symptoms and OAB, especially with the use of the mKMI score and OABSS.

Previously, evaluation of the association between menopausal symptoms and OAB was difficult owing to the lack of tools to assess these syndromes; however, the mKMI score and OABSS can now be used to assess these syndromes, and in the Chinese population, the mKMI score and OABSS have been used to assess menopausal symptoms and OAB, respectively [10,12].

The present study showed a significant association between menopausal symptoms and OAB. This association has been reported in a previous cross-sectional study [14]; however, the participants were nurses. Additionally, the previous study showed that the OABSS increases as the mKMI score increases. However, the previous study did not investigate the association between OAB and the frequency of sexual intercourse in the past 6 months but focused mainly on the OABSS and mKMI.

The present study identified five items of the mKMI that were associated with OABSS (sexual problems, UTI, vertigo, melancholia, and mood swings). Melancholia and mood swings are psychological symptoms of menopause, and the association between psychological stress and OAB has been reported in a previous study [15]. In the present study, UTI was found to have the strongest association with OAB statistically, which is in line with the findings of a previous study that showed an association between UTI and OAB [16]. Additionally, UTI is a symptom of menopausal symptoms. This indicates an association between menopausal symptoms and OAB. However, the association between vertigo and OAB remains unclear.

In the present study, an association between the frequency of sexual intercourse and OAB was identified; the frequency of sexual intercourse per month decreased as the severity of OAB increased. Several studies have shown an association between the frequency of sexual intercourse and OAB [17,18]. Milsom and Coyne found that OAB patients had worse sexual health than patients without urinary symptoms, based on a large cross-sectional survey [17]. Additionally, the negative effect of menopause on the frequency of intercourse has been reported [19]. Sexual activity may have a psychological, physiologic, or other such association with OAB. Studies have examined the association between sexual health and OAB [20,21]; however, the association has not been completely explained. Additionally, the above-mentioned studies have shown that both menopause and OAB can cause sexual problems. Owing to the limitations of cross-sectional studies, more studies on the relationship between OAB and sexual problems should be conducted in the future.

Recent studies have shown that age [5], BMI [22], DM [23], and heart diseases [24] are associated with the occurrence of OAB; Stewart found the prevalence of OAB with urge incontinence in patients with BMI ≥ 30 was 2.2 times higher than among patients with BMI < 24 [22]. Liu found the prevalence of OAB was 2.4 times greater in patients with DM duration > 10 years and age > 50 years [23]. Therefore, these were ruled out in the binary multivariate logistic regression analysis performed in the present study. Many previous studies involving large-scale cohorts have indicated that menopause is associated with obesity [25], DM [26], stroke [27], and heart diseases [28], and that menopause aggravates these diseases. Thus, menopausal symptoms and OAB have similar risk factors, and this may explain their association.

In the present study, 9.43% (38/403) of the participants experienced OAB (six women were excluded owing to use of hormonal drugs or traditional Chinese medicine as these may affect the evaluation of menopausal symptoms). However, this rate is below that reported in a previous population-based survey [1], and this may have occurred because of differences in the enrollment of women between that study and our study, and the limited number of women in our study.

The association between menopause and OAB can be explained physiologically. First, lack of estrogen is the main cause of menopausal symptoms. Estrogen helps increase cellular tropism in the epithelial layer of the vagina, urethra, and bladder, as well as in the trigone and puborectalis muscles owing to the presence of hormone receptors in these regions [29]. Therefore, estrogen can cause urinary symptoms by different mechanisms. Second, the prevalence of OAB increases after menopause. Third, menopausal symptoms and OAB have similar risk factors that involve emotion [30] and sexual function [17,19]. Psychological stress levels were higher in women with OAB than in healthy controls; therefore, stress levels are positively associated with the symptoms of UI. Finally, progestin receptors exist in the entire female genital tract. Progestin appears to have a detrimental effect on continence as it reduces the muscle tone of the urethra and bladder [29].

However, in a survey about the association between UI and menopause in 2013, previous studies were analyzed and not all were found to show an association between menopause and UI, but overall, menopause had little impact on the risk of UI in general after adjusting for age, and longitudinal studies are necessary to clarify the risk factors [31]. However, the purpose of our study was to determine the association between menopausal symptoms and OAB, not menopause and OAB, because menopause is not the only cause of menopausal symptoms, as a previous survey result showed that lifestyle, menstrual status, race/ethnicity, and socioeconomic status influence these symptoms in women age 40–55 years [32]. However, further evidence is needed to support the findings of our study.

Cross-sectional studies have several potential limitations. Firstly, the sample of this survey was relatively small, and the power of test was relatively low. Secondly, this study had limitations of recall bias (such as menarche information and other information needing to be recalled) and selection bias, which limit all questionnaire-based studies, and these cannot be excluded. Therefore, other types of studies, such as cohort studies, should be performed to further determine the factors involved in menopausal symptoms. Patients who visited our menopause clinic may have had more severe symptoms than other patients, possibly resulting in a selection bias. Research on a large number of menopause patients outside a hospital setting is needed.

Menopause is experienced by most women, and menopausal symptoms are mainly caused by menopause. However, other factors, such as socio-economic status, lifestyle, and nutrition may play a role in menopausal symptoms [33]. Menopausal symptoms are the external manifestation of menopause, but its severity varies among women. Not all women experiencing menopause will develop OAB, but women with severe menopausal symptoms may be at greater risk of developing OAB.

## Conclusions

In conclusion, menopausal symptoms may be closely associated with OAB, and the prevalence of OAB may be high in women with severe menopausal symptoms. Additionally, sexual activity may be associated with the severity of OAB. There is currently no tool for appropriately predicting or evaluating the risk of OAB occurrence, and the questionnaire used in the present study may be helpful in this regard. Additionally, the prevalence of OAB may be reduced by relieving the menopausal symptoms.

## Supporting Information

S1 Questionnaire (DOCX)
