# Supplementary material for: Association between Menopausal Symptoms and Overactive Bladder: A Cross-Sectional Questionnaire Survey in China
Source: PLoS One. 2015 Oct 8;10(10):e0139599. doi: 10.1371/journal.pone.0139599 (PMC4598107; doi:10.1371/journal.pone.0139599)
Supplement: S1 Questionnaire — (DOCX) [file pone.0139599.s001.docx]

date □□□□ year □□ month □□日 first visit √

The questionnaire of menopause health

Number:

1. Basic information

1). Name: 2. Birthday:

3).degree of education:

4). Nationality:

5). phone number: e-mail address:

6). Professionals:

| (1). State organizations, party and mass organizations, enterprises and institutions, |
| --- |
| (2). Professionals, (3)Staff and related personnel, |
| (4). Commercial, service personnel, |
| (5). Agriculture, forestry, animal husbandry and fishery, water conservation, production personnel, |
| (6). Production, transportation equipment operators, and related personnel, |
| (7). Soldier, |
| (8). Others, |

7).the monthly income in last year:

2. past medical history and marriage history:

1).smoking history(past/now) (1)now (2)never (3)have a past history

Passive smoking history (1)yes (2)no

2).chronic diseases(past/now):

| Diseases | Years(confirmed) | Formal treatment | Diseases | Years(confirmed) | Formal treatment |
| --- | --- | --- | --- | --- | --- |
| 1. hypertension□ | □□□□ | yes□ no□ | ⑧ myoma of uterus□ | □□□□ | yes□ no□ |
| 1. diabetes mellitus□ | □□□□ | yes□ no□ | ⑨benign ovarian tumor□ | □□□□ | yes□ no□ |
| 1. osteoprosis□ | □□□□ | yes□ no□ | ⑩benign breast tumor□ | □□□□ | yes□ no□ |
| 1. hyperlipoidemia□ | □□□□ | yes□ no□ | cervical cancer/endometrial cancer/ovarian cancer □ | □□□□ | yes□ no□ |
| 1. Coronary heart disease□ | □□□□ | yes□ no□ | breast cancer□ | □□□□ | yes□ no□ |
| 1. stroke□ | □□□□ | yes□ no□ | others__________ | □□□□ | yes□ no□ |
| 1. cervical spondylosis□ | □□□□ | yes□ no□ | others__________ | □□□□ | yes□ no□ |

3). who did you live with last year?(multiple choice)

(1)husband (2)children (3)parents (4)mother and father in law (5)grandchildren 6)live alone

4). menoarch: last menstruation date:

(hysterectomize surgery date:

Surgical removal of the bilateral ovaries date: )

5). marital status:

(1) first marriage (2)divorced (3)remarriage (4)unmarried(have sexual activity) (5)unmarried(without sexual marriage) 6)widowed

6 pregnancies in total(including abortion, odinopoeia, spontaneous labor, caesarean section, ectopic pregnancy) □□

Term birth□□, spontaneous labor□□ caesarean section□□

7). frequency of sexual life in last six months:

(1)≥8times per month (2)4-7times per month (3)≤3 times per month (4)none

3. basic health status:

1). height□□□cm; weight: _ kg BMI:_ waist: _ cm hipline: _ cm

2). Basic heart rates(p): _ bmp, blood pressure:_

4. menopause syndrome evaluate

1). Frequency of menstruation in last 12 months:

(1)none(answer 2td question) (2)less than 12times(answer 3td question) (3)regular or ≥12times(answer 3td question)

2).Reason for menopause:

(1). Natural menopause (2).radiation (3). Medical drugs (4).surgery---if Surgery remove the bilateral ovaries? Yes_ no_ don’t now_

3).regardless of the menstruation status, are/were you aware of the menopause syndrome?

Yes_ no_

4). The duration of menopause syndrome: □year□month□

5)**．**The m**Kupermann score below can evaluate the symptom of menopause syndrome**，please fill the score in the corresponding grid according to your symptoms, please put it on the front of**“×”**，then we can calculate it correctly。

| **symptoms score** | **0** | **1** | **2** | **3** | Weighted score |
| --- | --- | --- | --- | --- | --- |
| Hot flashes/sweating | no | <3times/day | 3-9times/day | 10times/day | **×4** |
| Paresthesia | no | Occasionally | often, can tolerate | Feeling of cald, hot, pain disappear | **×2** |
| Insomnia | No | Occasionally | often, can tolerate | severe，affecting work and life | **×2** |
| Irritable | No | Occasionally | often, can tolerate | severe，affecting work and life | **×2** |
| Depression | No | Occasionally | often, can tolerate | severe，affecting work and life | **×1** |
| **Dizziness** | No | Occasionally | often, can tolerate | severe，affecting work and life | **×1** |
| **Fatigue** | No | Occasionally | often, can tolerate | severe，affecting work and life | **×1** |
| Stiff (courbature) | No | Occasionally | often, can tolerate | severe，affecting work and life | **×1** |
| **Headache** | No | Occasionally | often, can tolerate | severe，affecting work and life | **×1** |
| **Palpitation** | No | Occasionally | often, can tolerate | severe，affecting work and life | **×1** |
| Formication | No | Occasionally | often, can tolerate | severe，affecting work and life | **×1** |
| Sexual dysfunction | No | Sexual desire lower | often, can tolerate | Without sexual desire or sexual life | **×2** |
| Urinary tract infection | No | Occasionally | >3times/day, can self cure | >3times/day, need medicine | **×2** |

**mKMI score： （calculate by doctors or nurse）**

6). Attitude to MPS:

(1).frankly accept (2). a few worries (3). avoid(don’t think about it) (4).boredom

7).have you used sex hormone(Estrogen, progesterone and androgen, birth control pills, and other replacement drugs) after 35 years old?

(1)yes (2)no（answer the 9td question）

8).reasons for use sex hormone:

（1）. functional uterine bleeding （2）. Osteoporosis （3）.MPS （4）. Endometriosis （5）. Adenomyosis （6）. Urinary tract infection （7）.others——

9). have you got food guide of MPS? Yes_ no_

10). have you got medicine treating of MPS: yes_ no_(jump the question below)

11). the medicine history:

| Drugs | doses | Began time | End time | Still use now |
| --- | --- | --- | --- | --- |
|  |  |  |  |  |
|  |  |  |  |  |

5. exercises evaluate:

1). exercises habit(multiple question):

(1). Walking (2). Jogging (3)speed walking (4)dancing (5)swimming (6)bodybuilding exercises (7)ball games (8)bicycle (9)others

2)≥3imes/week yes_ no_

≥30minutes/time yes_ no_

3). heart bpm≥170-age after exercises yes_ no_ don’t test it_

6. urinary incontinence evaluate:

The OABSS **score below can evaluate the symptom of OAB**，please fill the score in the corresponding grid according to your symptoms. the total score is your finally result.

| Question | symptoms | frequency | Score |
| --- | --- | --- | --- |
| 1. Daytime frequency | How many times do you typically urinate from waking in the morning until sleeping at night? | ≤7 | 0 |
|  |  | 8-14 | 1 |
|  |  | ≥15 | 2 |
| 2. Nighttime frequency | How many times do you typically wake up to urinate from sleeping at night until  waking in the morning? | 0 | 0 |
|  |  | 1 | 1 |
|  |  | 2 | 2 |
|  |  | ≥3 | 3 |
| 3. Urgency | How often do you have a sudden desire to urinate, which is difficult to defer? | Not at all | 0 |
|  |  | Less than once a week | 1 |
|  |  | Once a week or more | 2 |
|  |  | About once a day | 3 |
|  |  | 2–4 times a day | 4 |
|  |  | 5 times a day or more | 5 |
| 4. Urgency incontinence | How often do you leak urine because you cannot defer the sudden desire to  urinate? | Less than once a week | 0 |
|  |  | Less than once a week | 1 |
|  |  | Once a week or more | 2 |
|  |  | About once a day | 3 |
|  |  | 2–4 times a day | 4 |
|  |  | 5 times a day or more | 5 |

**OABSS score： （calculate by doctors or nurse）**

7. test result:(filled by doctor or nurse)

1). Pelvic floor muscle function: Ⅰdegree Ⅱdegree

2). FPG：_mmol/L， 2-hour post-meal blood glucose­_mmol/L， Hba1c_

3). TG mmol/L　　 TCHO mmol/L

HDL－C mmol/L　 LDL－C 　 mmol/L

4). Thyroid function: T**_３_**　　 nmol/L T**_４_** 　　　　nmol/L

FT**_３_**　　　　pmol/L FT**_4_**　pmol/L　　　 TSH uIU/mL

5). LH mIU/ml FSH mIU/ml E2 pg/ml PRL ng/ml

T ng/ml P ng/ml

6)BMD: T score

Standard SF-36 form was omitted.

Thank you!
